# Supplementary material for: Splice-Junction-Based Mapping of Alternative Isoforms in the Human Proteome
Source: Cell Rep. Author manuscript; Available in PMC 2020 Jan 15. (PMC6961840; doi:10.1016/j.celrep.2019.11.026)
Supplement: 2 [file NIHMS1546469-supplement-2.zip › DF1/PXD010154/Colon/Colon_11_MYCBP2_PAPSIFDQAAKPPSSLVHSPFVFGQPLSFQQPQLQSDR.pdf]

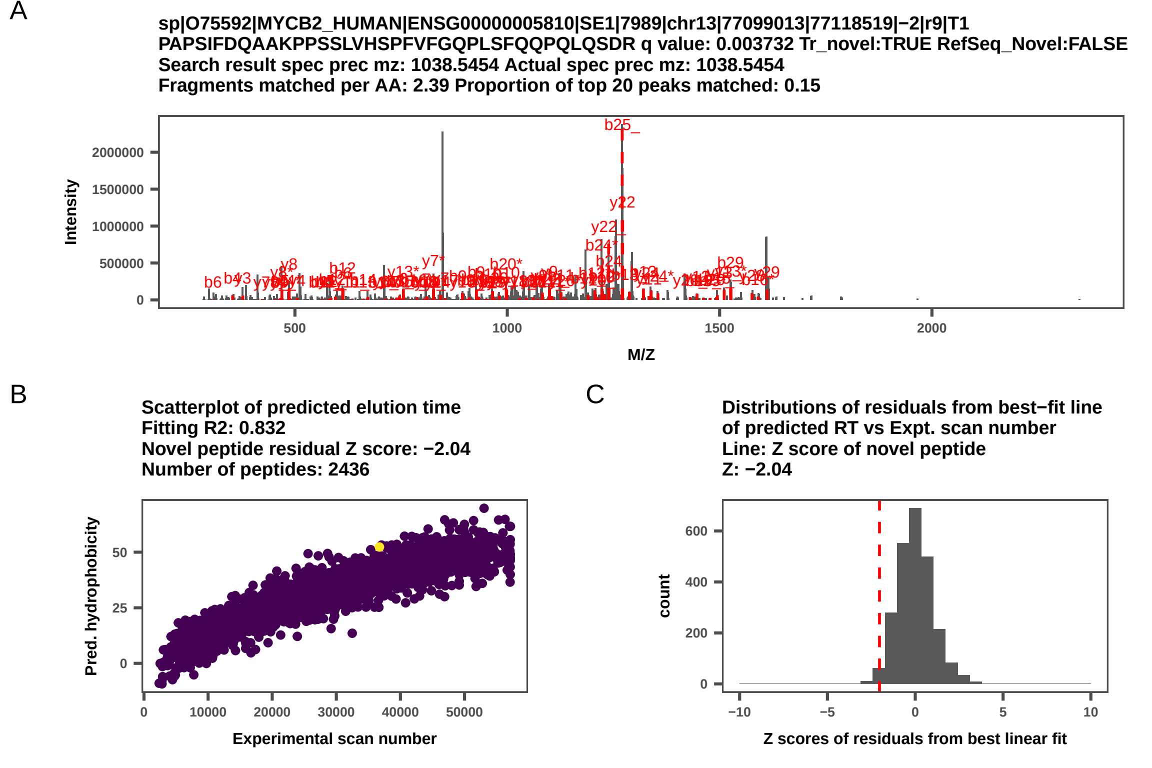
A
sp|O75592|MYCB2_HUMAN|ENSG00000005810|SE1|7989|chr13|77099013|77118519|−2|r9|T1
PAPSIFDQAAKPPSSLVHSPFVFGQPLSFQQPQLQSDR q value: 0.003732 Tr_novel:TRUE RefSeq_Novel:FALSE
Search result spec prec mz: 1038.5454 Actual spec prec mz: 1038.5454
Fragments matched per AA: 2.39 Proportion of top 20 peaks matched: 0.15
Intensity
2000000
1500000
1000000
500000
0
b25_
y22
b24
M/Z
500
1000
1500
2000
B
Scatterplot of predicted elution time
Fitting R2: 0.832
Novel peptide residual Z score: −2.04
Number of peptides: 2436
Pred. hydrophobicity
50
25
0
Experimental scan number
0
10000
20000
30000
40000
50000
C
Distributions of residuals from best−fit line
of predicted RT vs Expt. scan number
Line: Z score of novel peptide
Z: −2.04
count
600
400
200
0
Z scores of residuals from best linear fit
−10
−5
0
5
10
